# Hemichorea after hypoglycemic episodes with negative MRI findings in an elderly woman with poorly controlled type 2 diabetes mellitus: a case report

**DOI:** 10.1186/s12883-019-1334-2

**Published:** 2019-06-15

**Authors:** Eriko Matsushima, Hiroshi Shiota, Kentaro Watanabe, Yuichiro Otsuka, Midori Yamana, Suguru Yamaguchi, Fujiko Egashira, Satoshi Kamei, Hisamitsu Ishihara

**Affiliations:** 1 Division of Diabetes and Metabolic Diseases, Department of Medicine, Nihon University School of Medicine, 30-1 Oyaguchikami-cho, Itabashi-ward, Tokyo, 173-8610 Japan; 2 Division of Neurology, Department of Medicine, Nihon University School of Medicine, 30-1 Oyaguchi Kami-cho, Itabashi-ku, Tokyo, 173-8610 Japan

**Keywords:** Diabetic chorea, Elderly woman, Hypoglycemia

## Abstract

**Background:**

Diabetic chorea appears during the course of poorly-controlled diabetes. While chorea associated with diabetes mellitus usually occurs during hyperglycemic episodes, hypoglycemia can also cause diabetic chorea. Brain magnetic resonance imaging (MRI) is useful for evaluating the pathogenesis of diabetic chorea. However, several diabetic chorea cases have reportedly not shown abnormal high-intensity in the putamen and striatum on T1-weighted images.

**Case presentation:**

We report a 74-year-old woman who was admitted to our hospital for treatment of poorly-controlled type 2 diabetes mellitus. Intensified insulin treatment gradually normalizeed blood glucose, but on the 19th hospital day, after a blood glucose measurement of 49 mg/dL, she showed hemichorea of the left face, shoulder, arm and leg. MRI revealed no abnormalities of either the putamen or the striatum on T1-weighted images. She was treated with dopamine receptor antagonists, which alleviated her hemichorea symptoms and allowed discharge from the hospital. 1 year after the first hospitalization, she had to be readmitted because her glycemic control had markedly deteriorated. Glycemic control improved rapidly, and, because hemichorea did not recur, the dopamine receptor antagonists were stopped. 1 month later, however, hemichorea recurred. She resumed taking the dopamine receptor antagonists, resulting in immediate disappearance of the hemichorea.

**Conclusions:**

We herein describe a rare case of diabetes-associated hemichorea occurring after hypoglycemic episodes without abnormal high-intensity findings in the basal ganglia on T1-weighted images. The hemichorea relapsed with cessation of dopamine receptor antagonists. This case also underscores the importance of longitudinal assessment and treatment for hemichorea after hypoglycemic episodes, even in the absence of MRI findings, in elderly diabetic patients.

**Electronic supplementary material:**

The online version of this article (10.1186/s12883-019-1334-2) contains supplementary material, which is available to authorized users.

## Background

Chorea can be induced by structural, neurochemical, or metabolic disorders involving the basal ganglia. Diabetic chorea, a manifestation of the metabolic disturbance in the brains of patients with diabetes, appears during the clinical course of poorly-controlled diabetes. Most patients with diabetic chorea are reportedly Asian [1]. While chorea associated with diabetes mellitus usually occurs during hyperglycaemic episodes, hypoglycemia can also cause diabetic chorea [2–8]. Brain magnetic resonance imaging (MRI) is useful for evaluating the pathogenesis of diabetic chorea. Pathognomonic changes typical of diabetic chorea on brain MRI include abnormal high-intensity findings in the contralateral basal ganglia on T1-weighted images [9]. However, several diabetic chorea cases have reportedly not shown these abnormal high-intensity findings in the putamen and striatum on T1-weighted images [10–13].

Herein, we report an elderly woman with type 2 diabetes who showed hemichorea after hypoglycemic episodes without abnormal high-intensity on brain MRI. Furthermore, this patient experienced recurrence of hemichorea 1 year after the initial episode. To our knowledge, there have been no prior reports of patients showing diabetes-related recurrent hemichorea, initially occurring after hypoglycemic episodes, but without abnormal high-intensity findings in the putamen on T1- weighted images.

## Case presentation

A 74-year-old woman with type 2 diabetes mellitus was admitted to our hospital for management of poorly-controlled diabetes. Diabetes, diagnosed at age 49 years, was treated with insulin injections. She was taking insulin glargine 14 units, insulin aspart 24 units, sitagliptin 50 mg and metformin 500 mg, daily, for treatment of hyperglycemia before the first admission. She was also administered antihypertensive, lipid-lowering and antiulcer medications (nifedipine 40 mg, trichlormethiazide 1 mg, rosuvastatin 2.5 mg and famotidine 20 mg per day). She had undergone cataract and pelvic fracture surgeries at 63 and 71 years of age, respectively. She had no history of diabetic ketoacidosis, diabetic coma, severe hypoglycemic episodes, impaired renal function, hepatic dysfunction or central nervous system manifestations, including chorea, before the first admission. She had no history of either smoking or habitual alcohol consumption.

**Fig. 1:**
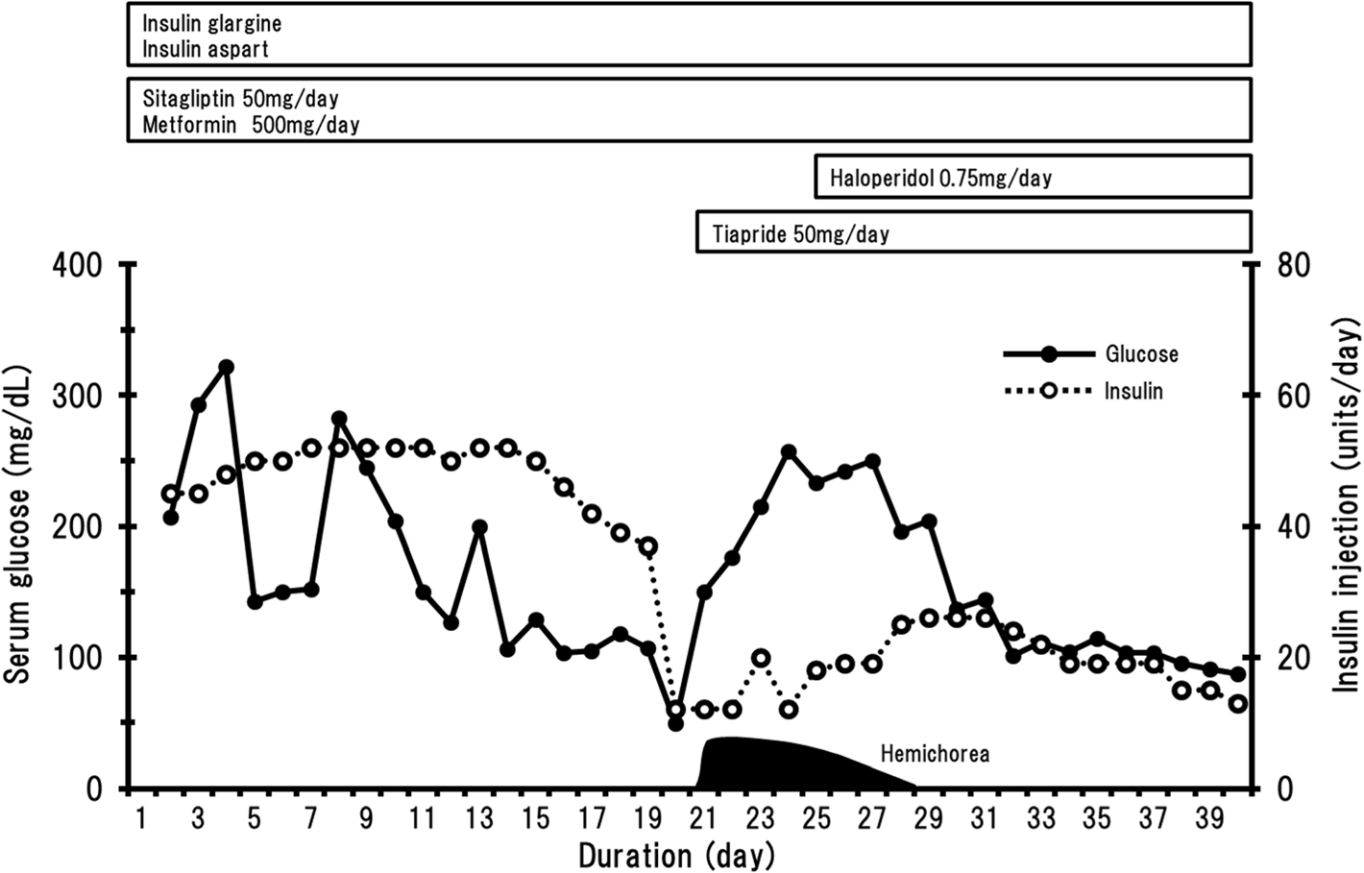
Clinical course during the first admission

This patient’s clinical course is presented in Fig. 1. No physical, including neurological, abnormalities were found. Glycosylated haemoglobin (HbA1c) and the serum glucose level on admission were 11.1% and 213 mg/dL, respectively. Intensive treatment with insulin injections was thus necessary. After several days, her glycemic control improved. The patient showed symptoms of hypoglycemia, such as palpitations and cold sweats, from a few days to 1 day before the first attack of hemichorea. Hypoglycemia was managed with glucose ingestion. The last symptomatic hypoglycemic episode during hospitalization appeared 1 day prior to the first hemichorea attack. Continuous glucose monitoring (CGM) was performed for daily evaluation of glycemic control, and the lowest serum glucose level measured during CGM was 49 mg/dl.

**Fig. 2:**
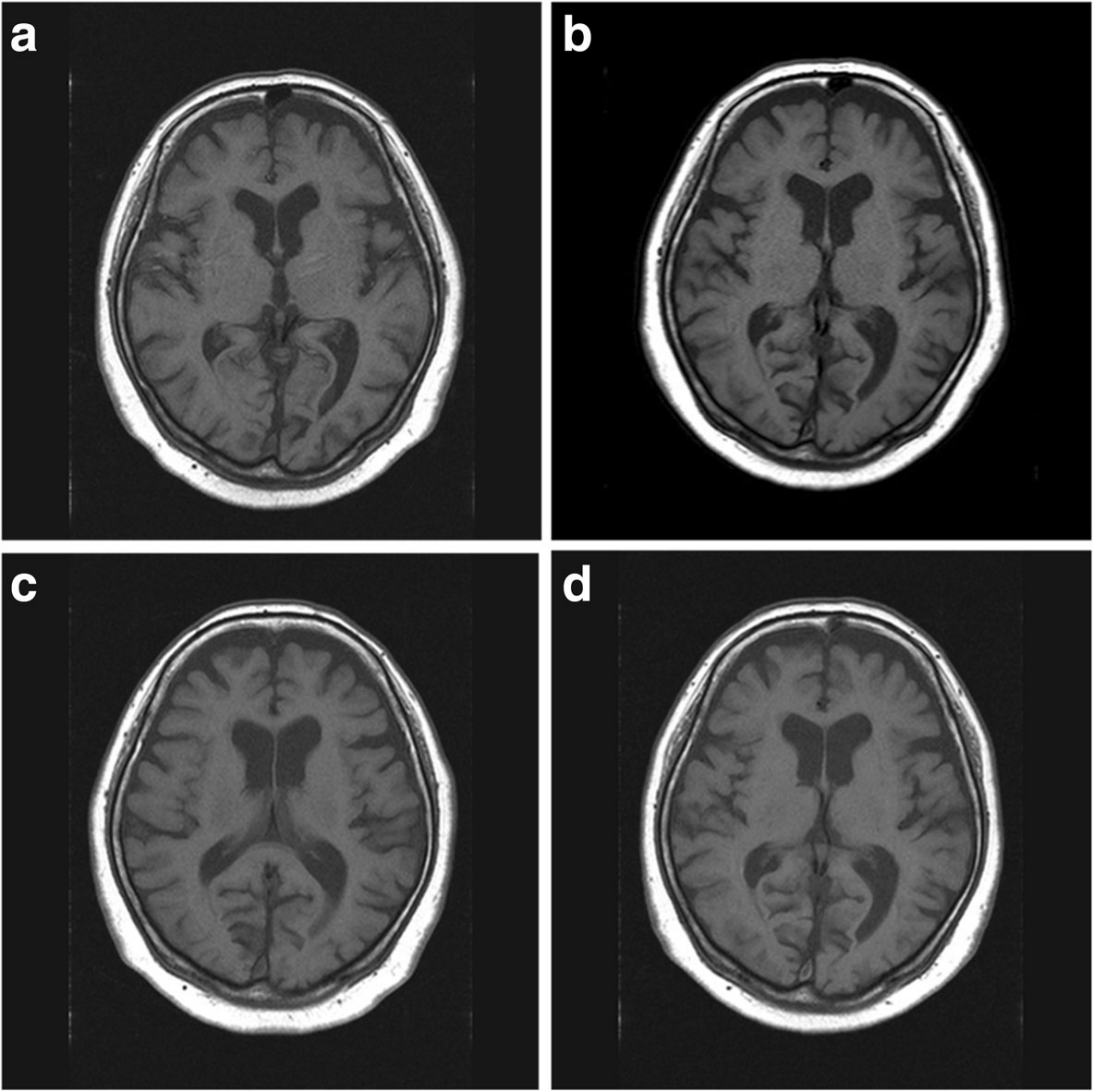
T1-weigted brain MRI scans. **a** on the 19th hospital day during the first admission. **b** at 1 month after discharge. **c** on admission for the second hospitalization. **d** at the time of hemichorea recurrence. There were no remarkable changes in basal ganglia on any of the T1-weighted brain MRI scans

On the 19th hospital day, sudden involuntary movements involving the left face, shoulder, arm and leg [see Additional file 1] were observed. These involuntary movements were exacerbated by stress, but diminished during sleep. No other remarkable neurological abnormalities were noted and there was no muscle weakness in either the upper or the lower limbs. A brain MRI scan was obtained during the involuntary movements. However, no abnormal high-intensity areas on T1-weighted images associated with the involuntary movements were detected in either the putamen or the striatum (Fig. 2a). In addition, there were no abnormal signal intensity findings in the putamen were on T2-weighted, FLAIR, or diffusion-weighted images. Neither the cerebral nor the carotid artery was stenotic on brain MR angiography imaging and carotid ultrasonography. She had no family history of diseases characterized by involuntary movements, such as Huntington’s disease. In addition, our patient’s thyroid functions were within normal range and serum thyroid autoantibodies were negative. Collagen disease antinuclear antibodies and antiphospholipid antibody were also negative. These data pointed away from Hashimoto encephalopathy or encephalopathy associated with collagen diseases such as antiphospholipid antibody syndrome. Mitochondrial encephalomyopathy, lactic acidosis, and stroke-like episodes (MELAS) seemed very unlikely because none of the typical clinical abnormalities associated with this disease (mental disorder, cardiomyopathy, or deafness, etc.) was present. In addition, infection- associated forms of hemichorea, such as Sydenham’s chorea, seemed very unlikely because our case had no history of infectious disease from several months before admission. Furthermore, the last symptomatic hypoglycemic episode had occurred 1 day before the onset of hemichorea, and the clinical course and examinations of our case showed no diagnostic indications of other etiologies of chorea unrelated to hypoglycemia- associated chorea. We thus speculate that the choreiform movements in this case, while not being accompanied by abnormal high- intensity in the contralateral putamen on T1-weighted brain MRI scans, were associated with hypoglycemic episodes.

Administration of dopamine receptor antagonists (tiapride 50 mg and haloperidol 0.75 mg per day, respectively) was initiated. During the first 4 days after the chorea attack, choreic movements occurred frequently and persisted for several hours, but the frequency of such involuntary movements decreased 5 days after the onset. Her involuntary movements showed improvement at 8 days after the onset of hemichorea. She was discharged on a maintenance regimen of dopamine receptor antagonists. A brain MRI scan was obtained 1 month after discharge, and again no remarkable changes were recognizable (Fig. 2b).

12 months after the first admission, she required readmission due to marked deterioration of glycemic control. Her HbA1c levels ranged from 10.0 to 12.0% during the time between admissions, but she had been free of hemichorea. She received intensive treatment consisting of food intake restriction and an increased insulin dosage, leading to improved glycemic control. No hypoglycemic episodes occurred during the second hospitalization. Follow-up brain MRI showed no remarkable changes (Fig. 2c). Dopamine receptor antagonists were discontinued before discharge. 1 month later, i.e. 38 days after administration of these medications had been stopped, the left-sided hemichorea recurred. Her serum glucose levels, measured with a self-monitoring device during the few days before the hemichorea re-manifested, had ranged from 187 to 231 mg/dL. The severity of hemichorea at recurrence was milder than that during the first episode. No brain MRI changes associated with hemichorea were detected at the time of recurrence (Fig. 2d). She resumed taking the dopamine receptor antagonists and the recurrent hemichorea **s**howed immediate improvement. In the 5 years, to date, since restarting the dopamine receptor antagonists she has experienced no further episodes of hemichorea.

## Discussion and conclusions

Our patient presented with hemichorea after hypoglycemic episodes without hyperintense lesions in the basal ganglia on T1-weighted brain MRI. She also exhibited recurrent hemichorea 1 month after discontinuing treatment with dopamine receptor antagonists.

Several reported cases were described as showing chorea with hypoglycemic episodes [2–8] (Table 1). These cases ranged in age from 45 to 80 years and their serum glucose levels from 20 to 66 mg/dL. Almost all of these prior cases were women being treated with insulin injections. Our case was typical in that she was elderly and required insulin therapy. However, the present case was atypical in that she had no abnormal brain imaging findings. Although remarkable changes on brain MRI have reportedly been absent in several patients with hyperglycemia-induced chorea, our case, to our knowledge, is the first shown to exhibit hemichorea without abnormal high-intensity findings in the basal ganglia on T1-weighted images obtained after hypoglycemic events.

**Table 1:** Case reports of chorea during or after hypoglycemic episodes in diabetic patients

No	Age (years)/ Sex	Serum glucose (mg/dL)	HbA1c (%)	Treatment of diabetes	Abnormal change in brain imaging		Recurrence of chorea	Reference
					CT	MRI		
1.	72/W^a^	n/a	n/a	n/a	–	n/a	–	2)
2.	59/W^c^	20	n/a	n/a	n/a	+	–	3)
3.	56/W	50	n/a	n/a	n/a	+	–	3)
4.	young/M	34–39	n/a	insulin	+	+	–	4)
5.	80/W	40	15.6	insulin	+	+	–	5)
6.	76/W	66	10.9	insulin	n/a	–	–	6)
7.	19/W	37–44	n/a	insulin	n/a	+	–	7)
8.	64/W^b,c^	n/a	n/a	n/a	+	n/a	–	8)
9.	This case (76/W)	49	11.1	insulin, OAD	–	–	+	

*n/a* not available, *W* woman, *M*: man, *CT* computed tomography, *MRI* magnetic resonance imaging, *OAD* oral anntihyperglycemic drug

^a^ This case had history of hypoglycemic comas

^b^ This case had history of admission of severe hypoglycemia

^c^ These two cases were treated with hemodialysis

Several possible pathophysiological mechanisms of cerebral damage associated with hypoglycemia have been suggested. One of these mechanisms might be anoxia due to cytotoxic edema resulting from glucose deprivation- induced sharp decreases in brain energy status and cellular ionic pump activity [3]. Another hypothetical mechanism, as with hyperglycemic chorea, might involve decreased blood flow in the basal ganglia and increased perfusion of the thalamus contralateral to the chorea, which also reportedly occurs in hypoglycemia [1]. Finally, it is also possible that depletions of gamma-aminobutyric acid and acetylcholine, which are required as an alternative energy source during hyperglycemia, began in the hyperglycemic period and then became more prominent during the hypoglycemia following treatment of hyperglycemia [1]. However, the association between the severity of brain damage and hypoglycemia-induced neurological symptoms remains unclear. Puente et al. reported hypoglycemia-induced brain damage to be less severe in rats that experienced recurrent moderate hypoglycemia than in those without prior recurrent hypoglycemia [14]. Schmidt et al. reported that short-term severe hypoglycemia induced by insulin per se caused no visible changes in either diffusion- weighted images or apparent diffusion coefficient maps on brain MRI [15]. These observations suggest that brain damage severities are not necessarily determined by the severity of hypoglycemia. Furthermore, a review of 42 prior reports indicated hypoglycemia-induced neurological abnormalities and brain imaging findings to be weakly associated with the depth or duration of hypoglycemia, neurological deficit severity, and imaging abnormalities [16]. Thus, neither brain imaging nor the severity of hypoglycemia is sufficient to diagnose the severity of hypoglycemia-induced neurological abnormalities.

Another important feature observed in the present patient was that hemichorea recurred 1 month after discontinuing dopamine receptor antagonists. Oh et al. reported that 7 out of 53 cases with hyperglycemia-induced chorea with MRI abnormalities showed recurrence from 2 months to 2 years after the first episode of chorea [1]. Thus, the severities of abnormalities found on brain imaging studies are not apparently related to relapse of diabetic chorea**.**

Several clinical issues warrant further examination in our case. First, the temporal association between hypoglycemia and hemichorea remains unclear. No symptomatic hypoglycemia was detected after either the initial onset of hemichorea or the recurrence, though it is possible for asymptomatic hypoglycemia to affect the frequency of hemichorea. Furthermore, other as yet unknown or unrecognized factors may have been present in our case. Infectious or autoimmune chorea, especially, could not be ruled out. Second, there may have been abnormal high- intensity areas in the putamen that disappeared prior to the MRI scans having been obtained. High signal intensity in basal ganglia lesions on T1-weighted images can reportedly occur before the onset of chorea, followed by resolution of this high signal intensity along with the improvement of chorea [1]. On the other hand, Shin and Ahn [17] reported that high signal intensity in the putamen was identified on T1-weighted brain images in 97.2% of patients with glycemic choreoballism associated with diabetes. Finally, neither brain SPECT nor PET imaging could be performed at the onset of chorea in our patient. Such images would likely have contributed useful information regarding the pathogenesis of hemichorea in this case.

In conclusion, we have described a rare patient showing hemichorea and hypoglycemia, without abnormal high- intensity in the basal ganglia on MRI, which recurred when dopamine receptor antagonists were discontinued. Our case indicates that we need to consider the possibility of involuntary movement after hypoglycemia being related to the hypoglycemia, even in the absence of abnormal high-intensity findings in the putamen on T1-weighted images, especially in elderly diabetic women treated with insulin. Also, the clinical course of our patient indicates that longitudinal assessment and treatment are necessary for elderly diabetic patients with a history of hypoglycemia- induced central nervous system manifestations, regardless of whether or not abnormal-high intensity findings are apparent on T1-weighted brain MRI.

## Additional file

Additional file 1: Hemichorea after hypoglycemic episodes. (MP4 1970 kb)

## Abbreviations

CGM: Continuous glucose monitoring
HbA1c: Glycosylated haemoglobin
MELAS: Mitochondrial encephalomyopathy, lactic acidosis, and stroke-like episodes
MRI: Magnetic resonance imaging
